# A feasibility study of scheduling a remission consultation in the management of patients treated for localized breast cancer

**DOI:** 10.1186/s40359-025-03393-6

**Published:** 2025-09-29

**Authors:** Sandrine Alonso, Sarah Kabani, Pascale Fabbro-Peray, Nadine Houédé, Luc Duwig

**Affiliations:** 1 Department of Biostatistics, Clinical Epidemiology, Public Health and Innovation in Methodology (BESPIM), CHU Nîmes, Univ Montpellier, Nîmes, France; 2 Institut de Cancérologie du Gard, CHU Nîmes, Univ Montpellier, Nîmes, France; 3 INSERM U1194, Montpellier Cancer Research Institute, Montpellier, France; 4 Department of Medical Oncology CHU Nîmes, Univ Montpellier, Place du Professeur Debré, 30029 Nîmes Cedex 09, France

**Keywords:** Body image, Cancer remission, Consultation, Psycho-oncology, Quality of life, Transition, Cancer survivors

## Abstract

**Purpose:**

To assess the feasibility of a remission consultation with on-going personalized care for patients treated for localized breast cancer entering a monitoring phase, while collecting quality of life data for one year. The primary objective was to evaluate the rate of acceptance of the remission consultation and, in the group who accepted, evaluate the change in quality of life using the EORTC QLQ-C30 over time. The secondary objectives were to evaluate change over time of the patient’s perception of body image via the BIS score, change over time in psychological distress score on a visual analog scale, time until return to work, and to describe the characteristics of patients according to the management methods chosen.

**Methods:**

Patients in remission from non-metastatic breast cancer were invited to a one-hour consultation interview to assess psychological condition and identify personalized supportive care needs. Various psychological follow-up options were offered depending on patient needs (e.g., anxiety or depression; fear of recurrence; or difficulty reconnecting with family, friends or at work). Patients were followed up at 3, 6 and 12 months after the remission consultation.

**Results:**

Of the 69 patients included, 50 (72% [95%CI 60–83]) accepted the remission consultation with a psychotherapist, with 27 opting for further follow-up. There was a significant improvement in patients’ overall quality of life (*p* = 0.01), physical functioning (*p* = 0.002) and body image perception (*p* = 0.007) over the study. Symptoms (pain, fatigue and dyspnea) significantly decreased over time. The psychological distress score halved, but was not statistically significantly different. The proportion of women returning to work increased over time.

**Conclusion:**

This study evaluated acceptance rate of remission consultations and the change in patients’ quality of life, well-being and reintegration over one year following the consultation. These results suggest that a remission consultation is feasible and would be well-received by a considerable number of patients. Offering a consultation at this stage represents a first step towards a better psychological management following cancer. These preliminary results require confirmation in a multicenter study with a randomized design.

**Trial registration number:**

NCT02740491, registered 14 April 2016.

**Supplementary Information:**

The online version contains supplementary material available at 10.1186/s40359-025-03393-6.

## Background

In parallel with therapeutic progress, cancer care optimization is essential, in the form of a personalized accompaniment and considering all the patient’s physical, psychological and social requirements [1]. Furthermore, cancer patients must be fully implicated in their own care [2]. An approach ensuring the continuity of care at different stages should be implemented, starting from the suspicion or diagnosis of cancer, continuing throughout systematic treatment (surgery, radiotherapy, chemotherapy) and beyond, during monitoring and follow-up [3]. Personalized post-cancer care programs should integrate supportive care and psycho-medico-social accompaniment [1, 3].

The incidence of breast cancer in France is over 50,000 cases per year and increasing by 0.6% each year, whilst the mortality rate is decreasing by 1.3% [4]. The total number of breast cancer survivors is therefore increasing. The period after breast cancer demands a mental adjustment from patients as they transition from patients to survivors. The VICAN 5 study [5] showed that 32.5% of patients report persistent degradation in their psychological quality of life, with many suffering from anxiety disorders and depression five years after cancer diagnosis. Certain risk factors for psychological distress following breast cancer include younger age, living alone, in poor, isolated, socio-economic conditions, and with a previous history of psychopathological disorders [6]. Other long-term problems include post-traumatic disorder, Lazarus syndrome (feeling out of sync with friends and family [7], or Damocles syndrome (constant fear of relapse) [8], and a fundamental modification of self-identity [9]. There is a termination in the supportive, nurturing effect of treatment and close ties with the medical profession, and this whole structure collapses once monitoring begins, leaving a feeling of abandonment [10]. Younger patients (< 46 years) in particular cite their disappointment with support tapering off or fading away, as they have many more years ahead of them [11]. Remission can confuse the patient’s identity because she feels neither completely ill nor fully recovered. Giving the patient chance for closure via a remission consultation may give her the opportunity to consolidate her feelings and move forwards, at her own rhythm.

The mental effort to recover from the trial of cancer presents a substantial burden, with wider implications both to the patient and their family [12]. Body image is an important element in cancer survivors. Indeed breast cancer and its treatments may considerably affect the patient’s image of her body, whether she has undergone surgery, radiotherapy, chemotherapy or hormone therapy [13, 14]. These body changes have negative impacts on the patient’s confidence and self-esteem. The post-cancer paradox consists of relief and satisfaction of regaining one’s health accompanied with a sudden feeling of vulnerability and the fear of a relapse, sometimes associated with a feeling of abandonment [15]. These emotional sequelae are associated with a poorer quality of life, greater dissatisfaction regarding care, an increase in the risk of non-compliance with treatment, a risk of unequal access to care, notably supportive care and post-cancer rehabilitation care (especially physical activity or dietary) and a delay in returning to work [13].

We hypothesized that a “remission” consultation, offered at the end of treatment alongside optional psychological accompaniment, may have a beneficial impact on these patients’ quality of life and body image perception. The aim was to accompany patients in the intermediate stage between somatic remission and psychological healing during their return to normal life. The accompaniment should lead to overall better reintegration of patients into their private and social lives. The primary outcome of this study was to assess the acceptance rate of the remission consultation and, among those who accepted, to evaluate the change in quality of life over one year following the consultation. This included assessing feasibility of implementing such consultations and collecting quality of life data for one year. Secondary outcomes were evaluating the change of body image and psychological distress over one year following the consultation, assessing return to work, and describing the characteristics of patients based to the management methods chosen.

## Methods

This was a prospective monocentric, pilot study. The study was approved by the French Commission Nationale de l’Informatique et des Libertés (number 1931126 v 0), is registered on clinicaltrials.gov (NCT02740491) and was performed in accordance with the Declaration of Helsinki.

### Participants

Participants were prospectively recruited from the Medical Oncology department of Nîmes University Hospital. Eligible participants were those > 18 years old receiving care for localized breast cancer having completed adjuvant treatment (chemotherapy, radiotherapy) within the previous 9 months. As this was a non-interventional study, signed consent was not required under the French Jardé law. However, a non-opposition letter detailing the study purpose was provided to eligible patients and patient data were only included if they did not return the non-opposition form. Patients were excluded if they had metastatic disease. Any patient experiencing relapse during the 12 months of follow-up was removed from the study. Patients were followed-up for 12 months.

### Remission consultation

At the end of their treatment, following the final consultation with their referring oncologist, patients were invited to participate in a “remission” consultation with a psychotherapist. The remission consultation lasted around one hour and was structured in two parts: the first focused on assessing the patient’s psychological state, while the second involved developing a personalized follow-up plan. First, patients were asked to recount their cancer journey, in their own words: “How did she react to the news? How did the people around her (family, friends, spouse, children, etc.) react? Is she supported? Has she stopped working or returned to work? How does she feel today? Does she have any specific needs? Is she still undergoing treatment (especially hormone therapy), and are there any side effects?”. The need for supportive care other than psychological support could then be determined: pain management, dietetics (weight gain, for example, under hormone therapy), adapted physical activity, intimacy/sexuality (vaginal dryness, dyspareunia, etc., with a gynecologist if necessary), sophrology, music therapy, socio-esthetic care, and information on post-breast cancer spa treatments. The patients were provided with information on other supportive services (e.g., oncology coordinating nurse) and organizations (e.g., Espace de rencontres et d’information, Ligue contre le cancer) and helped to make necessary appointments. Finally, the patient’s psychological state was determined: anxiety, depression, fear of recurrence, difficulty finding her place in her relationship, family, work, friends, change in body image, loss of femininity, sexuality etc. The clinician then recommended psychotherapy follow-up according to presence of any of the following: anxiety or depression; fear of recurrence; not recognizing oneself (psychologically or because of body changes: mastectomy, breast reconstruction, weight gain); or difficulty in reintegrating into the family or couple, or reconnecting with friends or at work. Depending on the patient’s needs, psychological support was offered either with the psychologist who performed the remission consultation, a psychologist the patient had seen previously, or with a private therapist. Patients could opt out of further visits, or switch between the different types of follow-up.

### Data collection and outcome measures

Patient characteristics provided by the oncologist were recorded, even for those who declined to participate. Before or at the start of the remission consultation, patients were asked to complete self-administered questionnaires that assessed quality of life, body image, and psychological distress. During follow-up, these questionnaires were completed during routine consultations where possible, or they were sent by post at 3, 6, and 12 months after the remission consultation. Patients who did not return the mailed questionnaires were recontacted up to three times. The feasibility of implementing a remission consultation was assessed by calculating the acceptance rate among all patients to whom the consultation was offered. Among those who accepted, engagement in this process was evaluated through the rate of patients who completed self-administered questionnaires during follow-up up to 12 months after the remission consultation.

The change in patients’ quality of life assessed at baseline, 3, 6 and 12 months was measured using the EORTC Quality of Life Questionnaire-Core 30 (QLQ-C30) version 3 [16]. The QLQ-C30 is a widely used instrument, validated in French, and consists of both multi-item scales and single-item measures. It includes five functional scales (physical, role, emotional, social, and cognitive), eight symptom scales (pain, fatigue, nausea and vomiting, insomnia, appetite loss, constipation, diarrhea, dyspnea), a financial difficulties scale and a global health status/quality of life scale. A summary score was calculated according to [17], and all other scores were calculated according to the scoring manual [18]. Scores were interpreted according to the minimally important differences (MID) calculated for certain subscores in patients with advanced breast cancer, where improvements in functional scales were established as 7–10 points for physical functioning, 7–9 points for social functioning, 5 points for cognitive functioning, 10–14 points for global health and 8 points for fatigue [19]. More generally, the effects of changes of 5–10 have been classed as “a little”, 10–20 as moderate, and > 20 as very much [20].

The change in body image assessed at baseline, 3, 6 and 12 months was measured using the Body Image Scale (BIS). The BIS is a 10-item questionnaire assessing negative sentiments towards body image in cancer patients on a 0 (not at all) to 3 (very much) scale; it is complementary to the QLQ-C30, is validated in French [21] and has been shown to be appropriate in a breast cancer population [22]. A cut-off of 10 points has been established as a predictor for psychological distress [23]. Change in psychological distress from baseline was measured on a 0-100 visual analogue scale (VAS) at 3, 6 and 12 months [24].

The date until return to work after the last radiotherapy or chemotherapy treatment was recorded for patients undergoing the remission consultation. To estimate the time until return to work, patients who did not return to work during the follow-up period were censored at their last known follow-up date or at the end of the study period and patients who had returned to work prior to the remission consultation were excluded.

Patient characteristics including age, living alone, living with dependents, social isolation, precarious socio-economic situation (unemployment or job loss), medical and psychiatric comorbidities, physical sequelae, untreated symptoms, and psychosocial unmet needs during treatment, were described and compared according to the management methods chosen at the end of the consultation. Comparisons were performed only for management methods applied to at least five patients.

### Sample size

This was a feasibility study and therefore a sample size calculation was not possible. It was considered that 50 patients would be representative of the patient population. Patients thus formed a convenience sample.

### Statistical methods

All statistical analyses were performed using SAS© (SAS Institute, Cary, NC, USA) version 9.4. Continuous variables were reported as means with standard deviations or medians with 25th and 75th percentiles, while qualitative variables were presented as frequencies with percentages. Student’s t-test or the Wilcoxon-Mann-Whitney test (depending on the distribution of continuous variables), as well as Chi-square or Fisher’s exact test, were used to compare the characteristics of patients who accepted or declined the consultation and those for whom different follow-up options were chosen by the psychologist during the consultation (among the most frequently observed, *n* > 5). The rate of patients accepting the remission consultation was presented with a 95% confidence interval (95% CI), calculated using the exact binomial Clopper-Pearson method. The longitudinal change of the QLQ-C30, BIS, and VAS psychological distress scores was tested using the Friedman test. When the Friedman test was significant, pairwise comparisons of differences between time points were performed using the Wilcoxon signed-rank test, with false discovery rate (FDR) correction applied for multiple comparisons. For the median differences between time points, a 95% confidence interval was estimated using the bootstrap method. Despite the small sample size and slight deviation from normality in the data, complementary analyses were performed using multivariate linear mixed models to assess the change of the QLQ-C30 summary score, BIS score, and VAS psychological distress scale over time, adjusting for potential confounders identified in univariate analysis (at a 0.2 significance threshold; all factors presented in Table 1 were tested). The time to return to work was described, and also estimated using Kaplan-Meier method along with the cumulative proportion of patients returning to work at 3 months, 6 months and 12 months with their 95% confidence intervals. All statistical tests were two-sided, with a significance level of 0.05.

**Table 1:** Main characteristics of patients available at the remission consultation according to their required follow-up* (among the most frequently observed: *n* > 5). * In addition to the groups compared, 6 patients required other types of follow-up (3 private psychological or psychiatric, 2 hypnotherapeutic, 1 psychological), and 1 patient refused follow-up despite the clinician’s recommendation

Characteristics	Not required (*n* = 22)	Psychotherapeutic (*n* = 21)	*p*-value
Age (years)	61 [50–69]	51 [46–59]	0.09
BMI (Kg/m^2^)	25 [23–28]	25 [22–28]	1
**Level of study** Primary school High school College University (≤ 2 years) University (> 2 years)	1 (4%) 2 (9%) 10 (46%) 3 (14%) 6 (27%)	/1 0 1 (5%) 7 (35%) 1 (5%) 11 (55%)	0.42
**Professional domain** Farming Artisan, Traders, Entrepreneurs Freelance and Managerial Administrative/commercial/technical, Supervisors Employed Manuel Laborers Retired, Unemployed or Inactive	1 (4.5%) 3 (13.6%) 0 5 (22.7%) 8 (36.4%) 1 (4.5%) 4 (18.2%)	0 2 (9.5%) 3 (14.3%) 5 (23.8%) 9 (42.9%) 2 (9.5%) 0	0.37
Living alone	2 (9%)	9 (43%)	0.01
Dependent child	8 (36%)	10 (48%)	0.45
Lost job	1 (5%)	1 (5%)	1
Alcoholism history	4 (18%)	0	0.11
Smoker	2 (10%) / 2	0 /7	0.57
Depressive symptoms	0	5 (24%)	0.02
Current psychological follow-up	0	7 (33%)	0.004
Current psychiatric follow-up	0	0	-
Alopecia	16 (76%) /1	2 (10%)	< 0.001
Lymphedema	3 (14%)	4 (19%)	0.70
Persistent pain	15 (68%)	15 (71%)	0.82
Time since last treatment to consultation (days)	99 [49–125]	131 [55–213]	0.09

Data are mean (SD), medians [25th-75th], or number (%), /: missing data. BMI: Body Mass Index

## Results

### Enrolment and baseline characteristics

**Fig. 1:**
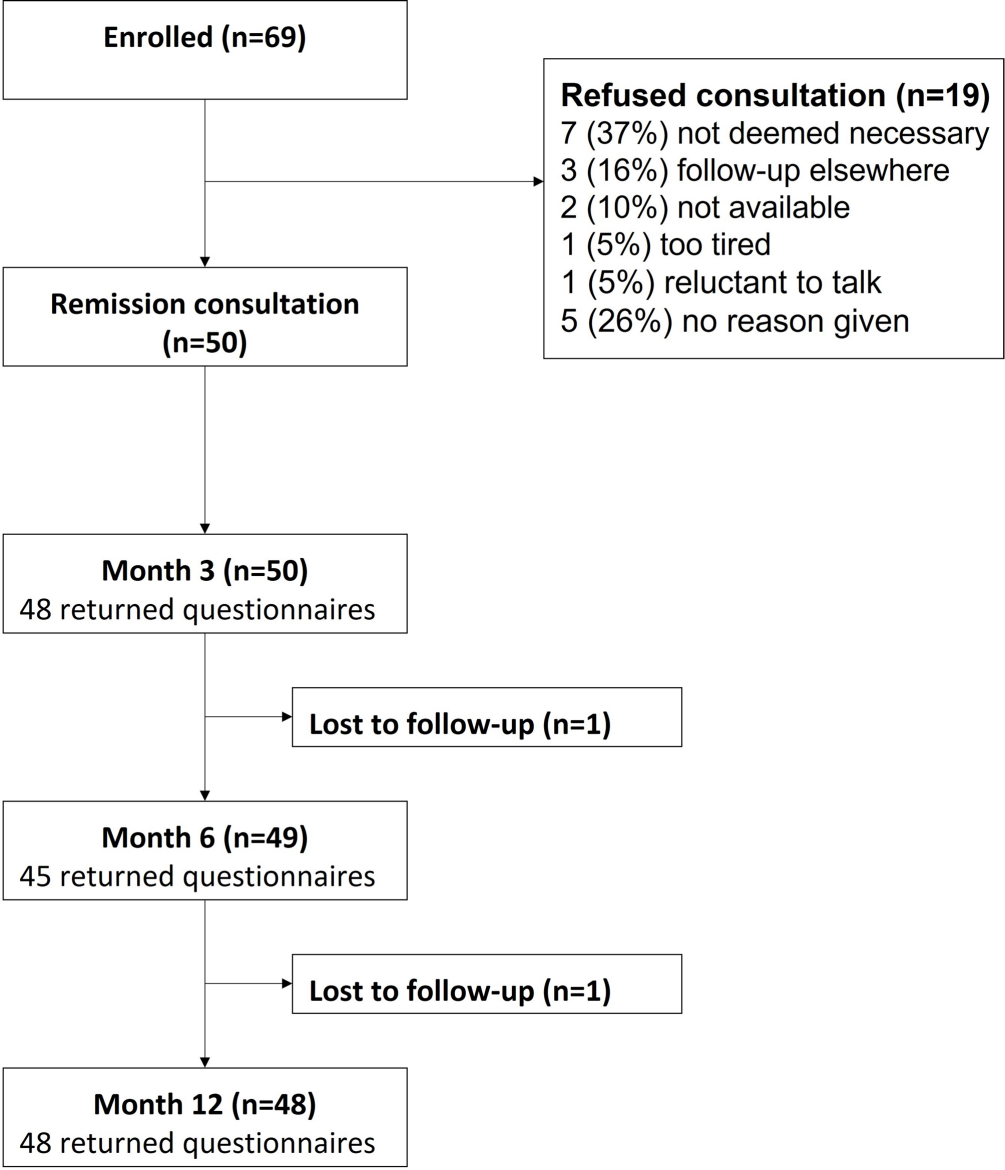
Flow diagram

Sixty-nine patients were enrolled between September 2016 and May 2018. The flow diagram is detailed in Fig. 1.

Table 2 shows the main characteristics of patients invited to participate in the remission consultation. Mean age was 56 (± 10) years and median BMI was 25 [22–28] Kg/m^2^: 25 (37%) had completed college level education and 25 (37%) had completed at least two years of university education. A quarter of participants (*n* = 17, 25%) lived alone and 5 (7%) had lost their job during illness. Nine (13%) had depressive symptoms and 11 (16%) were undergoing current psychological follow-up and none a current psychiatric follow-up. The most frequent somatic symptoms were alopecia in 28 (42%) patients and persistent pain in 41 (59%) patients. None of the patients relapsed during the study.

**Table 2:** Patient characteristics collected by oncologist after the end of treatment

	Total (*n* = 69)	Refused consultation (*n* = 19)	Accepted consultation (*n* = 50)	*p*-value
Age (years)	56 (10)	58 (10)	56 (11)	0.53
BMI (Kg/m^2^)	25 [22–28]	25 [22–32]	25 [22–28]	0.65
**Level of study** Primary school High school College University (≤ 2 years) University (> 2 years)	/2 5 (8%) 6 (9%) 25 (37%) 6 (9%) 25 (37%)	/1 4 (22%) 3 (17%) 7 (39%) 1 (5%) 3 (17%)	/1 1 (2%) 3 (6%) 18 (37%) 5 (10%) 22 (45%)	0.02
**Professional domain** Farming Artisan, Traders, Entrepreneurs Freelance and Managerial Administrative/commercial/technical, Supervisors Employed Manuel Laborers Retired, Unemployed or Inactive	/1 1 (1.5%) 9 (13.2%) 7 (10.3%) 15 (22.1%) 24 (35.3%) 4 (5.9%) 8 (11.8%)	/1 0 3 (16.7%) 0 4 (22.2%) 6 (33.3%) 1 (5.6%) 4 (22.2%)	1 (2%) 6 (12%) 7 (14%) 11 (22%) 18 (36%) 3 (6%) 4 (8%)	0.55
Living alone	17 (25%)	5 (26%)	12 (24%)	1
Dependent child	25 (36%)	3 (16%)	22 (44%)	0.03
Lost job	5 (7%)	2 (11%)	3 (6%)	0.60
History of alcohol abuse	7 (10%)	3 (16%)	4 (8%)	0.38
Smoker	5 (9%) /13	2 (13%) / 3	3 (8%) /10	0.21
Depressive symptoms	9 (13%)	2 (11%)	7 (14%)	1
Current psychological follow-up	11 (16%)	1 (5%)	10 (20%)	0.27
Current psychiatric follow-up	0	0	0	-
Alopecia	28 (42%) /2	8 (44%) /1	20 (41%) /1	0.79
Lymphedema	8 (12%)	1 (5%)	7 (14%)	0.43
Persistent pain	41 (59%)	8 (42%)	33 (66%)	0.07
Time since last treatment to oncologist consultation (days)	104 [36–191]	142 [80–190]	78 [28–194]	0.06

Data are mean (SD), medians [25th-75th], or number (%), /: missing data. BMI: Body Mass Index

### Acceptance

Fifty patients to whom the consultation was offered accepted to participate, with an acceptance rate of 72% [95%CI 60–83]. Among the 19 patients who refused, the reasons provided were most commonly that it was not considered necessary (*n* = 7, 37%), patients were already being followed up by someone else (*n* = 3, 16%), or the patient was not available (*n* = 2, 10%) (Fig. 1).

The rate of patients who completed at least one self-administered questionnaire was 49 (98%) at the remission consultation (the non-respondent subsequently completed questionnaires), 48 (96%) at 3 months, 45 (90%) at 6 months, and 48 (96%) at 12 months.

Patients who refused the consultation had less often completed at least two years of university education (*n* = 3, 17% vs. *n* = 22, 45%; *p* = 0.02) and were less likely to have a dependent child (*n* = 3, 16% vs. *n* = 22, 44%; *p* = 0.03). Persistent pain was observed more frequently in patients who refused (*n* = 8, 42% vs. *n* = 33, 66%), and the median time since last treatment to oncologist consultation was longer in this group (142 [80–90] days vs. 78 [28–194] days); however, these differences were not statistically significant.

### Characteristics according to follow-up

At the end of the remission consultation, 27 patients (54%) wished for further follow-up, with most (*n* = 21, 42%) choosing psychotherapeutic, followed by 3 (6%) choosing psychological or psychiatric (Supplementary Fig. 1). Twenty-two patients (44%) did not require further follow-up, and one patient refused follow-up despite the clinician recommending it.

The main characteristics of patients for the most frequent choices, namely those not requiring follow-up and those requiring psychotherapeutic follow-up, are presented in Table 1. Patients requiring psychotherapeutic follow-up most often lived alone (*n* = 9, 43% vs. *n* = 2, 9%; *p* = 0.05) and presented more frequent depressive symptoms (*n* = 5, 24% vs. 0; *p* = 0.02), and less frequent alopecia (*n* = 2, 10% vs. *n* = 16, 76%; *p* < 0.001). They also had higher uptake of current psychiatric follow-up (*n* = 7, 33% vs. 0; *p* = 0.004).

Change over time of quality of life, body image and psychological distress.

There was an increase in patient general quality of life over time (*p* = 0.01), the QLQ-C30 summary score increased from a median of 75 [64–90] at baseline to 83 [63–90] at 12 months, with a median change of 4.5 [1.3; 7.6] (*p* = 0.003). Physical functioning improved from 80 [67–87] to 87 [73–93] (*p* = 0.002), and cognitive functioning increased from 67 [50–100] to 83 [67–100] (*p* = 0.05), although neither met the MID for this population. Emotional functioning rose from 75 [42–92] to 83 [58–92] (*p* = 0.0180) with a median change of 8.3 [4.2; 16.7] (*p* = 0.007).

Improvements in functioning were accompanied by reductions in symptoms. Fatigue decreased from 44 [33–67] to 33 [22–56] (*p* = 0.0499), exceeding the threshold for a MID and classified as a moderate effect. Pain reduced from 33 [17–58] to 25 [17–58] (*p* = 0.0196), and dyspnea from 33 [0–33] to 33 [0–33] (*p* = 0.0209).

Body image also improved, with the BIS score decreasing from 11 [4–18] at baseline to 7 [3–14] at 12 months (*p* = 0.0070) and a median change of -3 [-4; -1] (*p* < 0.001). This change places the median below the cut-off for psychological distress associated with poor body image, with the number of patients above this cut-off reducing from 28 (57%) at baseline, to 20 (43%) at Month 3, down to 18 (38%) at Months 6 and 12. Psychological distress scores (VAS) dropped from 20 [0–50] at baseline to 10 [2–30] at 12 months, but this did not meet the cut-off for significance (*p* = 0.6495) (Table 3).

**Table 3:** Longitudinal change in quality of life (EORTC QLQ-C30), body image (BIS) and psychological distress (VAS) for patients who accepted the remission consultation

	Baseline (*n* = 49)	3 months (*n* = 48)	6 months (*n* = 45)	12 months (*n* = 48)	*p*-value
QLQ-C30					
Summary score Change:	75 [64–90] -	78 [62–91] 2.6 [0; 5.6]; *p* = 0.03	78 [62–91] 4.5 [2.5; 6.8]; *p* = 0.003	83 [63–90] 4.5 [1.3; 7.6]; *p* = 0.003	0.01
Physical Functioning Change:	80 [67–87] -	80 [67–93] 6.7 [0; 6.7]; *p* = 0.06	80 [67–87] 0 [0; 6.7]; *p* = 0.09	87 [73–93] 6.7 [0; 6.7]; *p* = 0.06	0.002
Role Functioning	67 [50–100]	67 [67–100]	83 [67–100]	67 [67–100]	0.50
Emotional Functioning Change:	75 [42–92] -	71 [50–92] 8.3 [0; 8.3]; *p* = 0.02	75 [58–92] 8.3 [0; 8.3]; *p* = 0.05	83 [58–92] 8.3 [4.2; 16.7]; *p* = 0.007	0.02
Cognitive Functioning Change:	67 [50–100] -	83 [67–83] 0 [0; 16.7]; *p* = 0.21	83 [67–100] 0 [0; 16.7]; *p* = 0.04	83 [67–100] 0 [0; 16.7]; *p* = 0.21	0.05
Social Functioning	83 [50–100]	83 [58–100]	83 [67–100]	100 [67–100]	0.32
Global health status	67 [50–83]	67 [50–83]	67 [58–83]	75 [58–83]	0.13
Fatigue Change:	44 [33–67] -	33 [22–61] 0 [-11.1; 0]; *p* = 0.20	33 [22–44] -11.1 [-11.1; 0]; *p* = 0.04	33 [22–56] -11.1 [-11.1; 0]; *p* = 0.09	0.05
Nausea Vomiting	0 [0–17]	0 [0–0]	0 [0–0]	0 [0–17]	0.40
Pain Change:	33 [17–50] -	33 [17–58] 0 [0; 0]; *p* = 0.35	33 [17–50] 0 [-8.3; 0]; *p* = 0.77	25 [17–58] -16.7 [-16.7; 0]; *p* = 0.35	0.02
Dyspnea Change:	33 [0–33] -	33 [0–33] 0 [0; 0]; *p* = 0.12	33 [0–33] 0 [0; 0]; *p* = 0.13	33 [0–33] 0 [0; 0]; *p* = 0.02	0.02
Insomnia	33 [0–67]	33 [0–67]	33 [0–67]	33 [0–67]	0.09
Appetite loss	0 [0–17]	0 [0–0]	0 [0–0]	0 [0–17]	0.38
Constipation	0 [0–33]	0 [0–33]	0 [0–33]	0 [0–33]	0.74
Diarrhea	0 [0–0]	0 [0–0]	0 [0–0]	0 [0–0]	0.87
Financial difficulties	0 [0–33]	0 [0–33]	0 [0–33]	0 [0–33]	0.74
Body Image Scale Change:	11 [4–18] -	7 [2–16] -1.5 [-2; -0.5]; *p* < 0.001	9 [2–18] -1.5 [-3; 0]; *p* = 0.06	7 [3–14] -3 [-4; -1]; *p* < 0.001	0.007
Psychological distress Change:	20 [0–50] -	20 [5–50] 0 [-9; 0]	15 [5–40] 0 [-10; 0]	10 [2–30] 0 [-10; 0]	0.65

Data are medians [25th-75th]. *p*-value: *p*-value for global change over time. Change: Median [95% Confidence Interval] change to baseline and FDR corrected *p*-value to Wilcoxon signed-rank test. n: number of patients who completed self-administered questionnaires

In sensitivity analysis, similar results were observed for changes in QLQ-C30 summary score, BIS, and psychological distress, both in unadjusted and adjusted linear mixed model (Supplementary Table 1).

### Time to return to work

Of the 50 patients accepting the consultation, 12 (25%) returned to work over the course of the study, at a median of 259 [112–365] days after last treatment (radio- or chemotherapy). Considering only the eight patients returning to work after the consultation, the median interval until return was 166 [118–299] days after remission consultation. Four patients (8%) returned to work prior to the remission consultation, rising to six (12%) at 3 months, nine (18%) at 6 months and 12 (25%) at 12 months. From the remission consultation (*n* = 46), the estimated proportion of patients who returned to work was 4% [1; 17] at 3 months, 11% [5; 25] at 6 months and 30% [11; 64] at 12 months (Supplementary Table 2).

## Discussion

In this study, we assessed the benefit of a remission consultation for patients with breast cancer on their quality of life and body image perception. Encouragingly, patients’ acceptance rate of the remission consultation was high, with around three quarters of patients accepting it. This rate is similar to that found in a study in patients with various cancers (59% breast cancer) undergoing screening for adjustment disorder [25]. The study authors found that 75% of patients at increased risk of adjustment disorder were willing to undergo a diagnostic interview, and 65% of those exhibiting adjustment disorder agreed to participate in a study testing personalized psychological treatment. The rate of psychological therapy uptake was also similar to another study on young adults with various cancers (average age 31) in which 56% of respondents had ever sought psychological counseling, with over 30% accessing this help at least four times [26]. Moreover, in an interview study of younger cancer patients, many participants reported that they would have appreciated knowing that mental health support was available earlier on, and that the onus falls on the patients to seek out help [11]. By offering this service within the same hospital structure, we removed this barrier. Altogether, this suggests that psychological therapy is appreciated by cancer survivors. The patients accepting the consultation had more often received previous psychological accompaniment and had more painful sequelae. There is therefore a slight bias because it was the most educated women who had already experienced psychotherapy who accepted this accompaniment. Interestingly, none of the variables tested by Van Beek et al. showed a difference in participants accepting or refusing psychological treatment for adjustment disorder [25]. Potential differences to be explored in subsequent studies of patients refusing the remission consultation include alcohol and tobacco consumption and rate of unemployment. Patients declining the proposed remission consultation claimed that they did not feel it was necessary, they had friends or family to support them or if they were unavailable.

The overall quality of life score increased significantly from 75 to 83 over the 12 months of follow-up. Emotional score and overall health score also increased. In parallel, two major symptoms, fatigue and pain, decreased in intensity. Fatigue, an extremely subjective symptom affected by emotional status with direct associations with quality of life, functioning, and coping [27], decreased from 44 to 33. Similarly, pain decreased from 33 to 25. The changes observed for physical and cognitive functioning did not meet the MIDs, whilst fatigue exceeded the MID established for patients with breast cancer. The greatest change seen in our study was for emotional functioning (change from baseline 8.3 points), however this subscore does not have a specific MID [28], although the size change would be classed as moderate according to the general cut-offs [20]. However, these MIDs should be interpreted with caution, as they were calculated from studies on patients with advanced cancer undergoing treatment rather than survivors in remission. Given the observed score fluctuations in our population and the lack of directly applicable thresholds for breast cancer survivors in remission, interpreting results using MIDs remains challenging.

These trajectories in quality of life mirror those found by Schmidt et al. in a cohort of breast cancer patients followed for five years [29]. In their study, pain and fatigue both reduced within the first year and then remained low. An Italian study on young breast cancer patients found that quality of life returned to normal levels 12 after surgery, although a large proportion of patients had high anxiety at this time point [30]. In contrast, a registry study of survivors of breast, cervical and colorectal cancer found that fatigue remained over-reported in cancer survivors even 15 years after diagnosis [31]. Schmidt et al. also found very high reported quality of life using the same scale used here, exceeding that of the reference population, which they ascribed to a recalibration of internal standards by cancer survivors. It is important to note that these studies assessed the natural progression of quality of life, unlike our study. In our study, pain was considered amongst the needs for other supportive care assessed during the remission consultation, and the patient was directed towards a pain specialist as required. Nevertheless, there is a small bias because a greater proportion of patients accepting the remission consultation reported pain than those refusing it (66% versus 42%), which likely increased their acceptance for the consultation session. The psychological distress measured showed a modest initial VAS of 20, which decreased by half by 12 months, however, this was not statistically significant. Nevertheless, it is tempting to make the association between this considerable reduction in psychological distress and the significant increase in the emotional score measured by the EORTC QLQ-C30 as proof of an improvement.

At the end of the remission consultation, 44% of the women did not require aftercare, and only one patient refused aftercare despite the clinician judging it necessary. Among the participants opting for aftercare, most chose the analytical psychotherapy proposed by the investigator who performed the remission consultation. This could possibly be explained by affectionate transference towards the psychoanalyst, established and firmly engrained early on, right from the remission consultation. Some patients likely chose to continue with the hospital psychoanalyst due to the cost of sessions in private practice in France, for which they would have been liable, whereas the hospital care was state-supported. For the patients opting for different follow-up, 8% were already undergoing therapy by a hospital or freelance mental health care professional and 4% had undergone brief therapy (hypnosis). It should be noted that 55% of the patients who had benefitted from psychotherapeutic accompaniment had followed further education courses (at least 2 years), compared with 27% who had not. This further confirms the tendency already observed among those who accepted the remission consultation or not.

This high level of women still requiring aftercare 12 months after remission corroborates the findings of Stanton et al. in 2005 [32]. Stanton et al. found that a majority of patients who had had breast cancer still had distress symptoms, which decreased in the 12 to 24 months following the end of treatment with a return to an overall psychological condition (distress, anxiety) and quality of life comparable to or even better than that of the general population. In our study, four patients (8%) were still receiving psychotherapeutic aftercare two years later. This shows that it can take considerable time to consolidate the trial of cancer and to regain the capacity of projecting oneself into the future.

Body image perception improved over time, with a decreased BIS score from 11 to 7 after 12 months, which indicates that patients had a better perception of their body image over time and made progress in the psychotherapeutic work. This change crosses the cut-off point for psychological distress of ≥ 10, although no MID has been calculated for this scale [23]. Indeed, the number of patients classed as having psychological distress dropped from 28 at inclusion to 18 by 6 months. A meta-analysis on younger breast cancer survivors showed that the type of surgery influenced appearance satisfaction, with adjuvant radiotherapy and chemotherapy both having negative impacts on body image [33]. Four patients in our study underwent breast reconstruction; one before the remission consultation. For the three other patients, the psychological follow-up probably influenced this reconstruction choice. Furthermore, these reconstructions may themselves have contributed to the patients’ better perception of their body image. Other studies on survivors have shown that patients undergoing mastectomies have increased body image concerns [14, 34]. A study on patients with early stage breast cancer found that body image was most severely affected within the first six months postoperatively, but remained poor for the following six months [35]. Few studies have looked at whether interventions can improve body image in breast cancer survivors, but those incorporating physical activity perform better than couples or sex therapy alone [33].

The return-to-work rate increased over the course of the study. We found that 8% of patients had resumed work prior to the remission consultation, rising to 25% at 12 months after the remission consultation. These rates appear modest in comparison with the published literature, but are an encouraging sign of the good physical and psychological health for these 25% of women. For 16% of women who resumed work after the remission consultation, the median time taken to resume work was 166 days after the consultation. These rates are considerably lower than found in a systematic review of studies applying various interventions (counseling and physical exercises) at the transition stage, where the return-to-work rate was over 75% [36]. The length of time until resuming employment was proportional to the extent of surgical intervention. A systematic review found that both general and role function and social function affected the likelihood of returning to work, although without difference to the delay of the return, and yet physical functioning had the greatest impact on return to work [37]. A more recent French study found a return-to-work rate of 42.2% after one year in a younger cohort (average age 48 vs. 56 in our study), with nearly a quarter of patients returning on a part-time basis [38]. The lower return rate may partly reflect the French social support system, which means patients are not under such financial constraints to work before they feel ready.

### Study limitations

This study had several limitations. The sample size was small, although 50 patients seemed sufficient for an observational pilot study to judge the feasibility of the remission consultation and subsequent management. Recruitment into the study was complicated by the limited consultation slots available by study investigators and the inclusion criteria that required patients to be enrolled within 9 months following final treatment. In future studies, more including oncologists will be necessary, and follow-up schedules optimized. The small sample size and the study design did not allow analyses leading to robust conclusions, thus the differing characteristics in the group of patients who accepted the remission consultation (higher education level, higher socio-professional profile and more dependent children), should be investigated in future studies. Moreover, treatment and tumor characteristics such as histological subtype and cancer stage were not collected in this study, yet they would likely influence outcomes; this information will be essential to record in future studies. We only considered quality of life and body image, whereas breast cancer patients also experience other problems such as anxiety and impaired sexual function. Moreover, the major bias of our study is the absence of a control group, preventing us from directly attributing the improvements observed to the remission consultation. This makes it challenging to disentangle the effects of the remission consultation from the natural recovery process or other external factors influencing quality of life. There are ethical implications of randomizing patients to a control group with no intervention, which could leave patients in distress. However, an active control group could be a more appropriate option, for example offering a final consultation with a non-specialist (general practitioner or nurse). In our study, the patients refusing the remission consultation could have later psychological follow-up if they wished. The benefit of a simple VAS for measuring psychological distress was the low burden for patients, however, more exhaustive and widely used questionnaires should be considered in future studies, such as the Psychosocial Distress Questionnaire-Breast Cancer or the 12-item General Health Questionnaire.

### Clinical implications

This study is a first step towards establishing a better psychological management after breast cancer. To the best of our knowledge, it is the only study that has attempted to measure the impact of a post-cancer consultation on patients’ quality of life and well-being, as well as their reintegration. Specifically, this consultation considers the specific “transition” period that is not classically targeted in clinical studies, but is an important moment presenting the opportunity to help guide patients towards their preferred therapy.

## Conclusion

Based on our positive preliminary results, we envisage proposing a remission consultation at our institution for women who have finished their treatment for localized breast cancer and entering into a monitoring phase for patients displaying signs of psychological distress. Coordination and organization between the hospital and the patient’s personal doctor would have to be carefully considered in order to monitor this new flow of patients. While our study aimed to assess the impact of a remission consultation, it is important to note that quality of life improvements observed over time are consistent with findings from studies tracking quality of life without specific interventions. This highlights the natural progression of quality of life and suggests that the changes observed in our study cannot be solely attributed to the remission consultation.

Nevertheless, our results are encouraging, warranting further studies with a multicentric design with a control group to confirm them the benefit of integrating a remission consultation with optional psychological follow-up for patients, as a future standard procedure in post-cancer management.

## Supplementary Information

Below is the link to the electronic supplementary material.

Supplementary Material 1

Supplementary Material 2

## Data Availability

All data from this study are accessible upon reasonable request to luc.duwig@chu-nimes.fr.

## Abbreviations

BIS: Body image scale
EORTC QLQ-C30: European organisation for research and treatment of cancer quality of life questionnaire-core 30
MID: Minimally important differences
VAS: Visual analogue scale
