# Supplementary material for: A feasibility study of scheduling a remission consultation in the management of patients treated for localized breast cancer
Source: BMC Psychol. 2025 Sep 29;13:1087. doi: 10.1186/s40359-025-03393-6 (PMC12482605; doi:10.1186/s40359-025-03393-6)
Supplement: Supplementary file 1 — Supplementary Material 1 [file 40359_2025_3393_MOESM1_ESM.docx]

Stopped follows-up

n=2

Hypnosis

N=2

Psychotherapy at hospital

N=12

Follow-up by a community psychologist or psychiatrist

N=4

Follow-up by a hospital psychologist

N=1

Stopped follow-up

n=2

Lost to follow-up

n=1

Hypnosis

N=2

Psychotherapy at hospital

N=14

Follow-up by a community psychologist or psychiatrist

N=4

Follow-up by a hospital psychologist

N=1

Hypnosis

N=2

Psychotherapy at hospital

N=17

Follow-up by a community psychologist or psychiatrist

N=4

Follow-up by a hospital psychologist

N=1

Hypnosis

N=2

Psychotherapy at hospital

N=21

Follow-up by a community psychologist or psychiatrist

N=3

Follow-up by a hospital psychologist

N=1

Lost to follow-up

n=1

Stopped follow-up

n=3

N=2

N=1

Remission Consultations (n=50)

Evaluation at Month 12

Baseline

Follow-up chosen

Evaluation at Month 6

Evaluation at Month 3
