# Supplementary material for: A feasibility study of scheduling a remission consultation in the management of patients treated for localized breast cancer
Source: BMC Psychol. 2025 Sep 29;13:1087. doi: 10.1186/s40359-025-03393-6 (PMC12482605; doi:10.1186/s40359-025-03393-6)
Supplement: Supplementary file 2 — Supplementary Material 2 [file 40359_2025_3393_MOESM2_ESM.docx]

Supplementary Table 1. Longitudinal change in Quality of life (EORTC QLQ-C30), body image (BIS) and psychological distress (VAS) for patients who accepted consultation: Unadjusted and Adjusted Estimates from Linear Mixed Models

|  | Unadjusted | | Adjusted | |
| --- | --- | --- | --- | --- |
|  | Estimates | p-value | Estimates | p-value |
| QLQ-C30 Summary Score | n=186 | 0.01 | n=182 | 0.01 |
| Estimated mean±se level at baseline / n | 73±2 /n=48 |  | 72±5 /n=47 |  |
| Estimated mean±se level / n |  |  |  |  |
| At 3 months | 76±2 /n=47 |  | 75±5 /n=46 |  |
| At 6 months | 77±2 /n=45 |  | 76±5 /n=44 |  |
| At 12 months | 77±2 /n=46 |  | 76±5 /n=45 |  |
| Estimated mean±se change from baseline |  |  |  |  |
| To month 3 | 2.8±1.5 | 0.06 | 3.1±1.5 | 0.05 |
| To month 6 | 4.7±1.5 | 0.002 | 4.9±1.5 | 0.002 |
| To month 12 | 3.9±1.5 | 0.01 | 4±1.5 | 0.01 |
| Body Image Scale Score | n=187 | 0.01 | n=183 | 0.001 |
| Estimated mean±se level at baseline / n | 12±1 /n=49 |  | 9±2 /n=48 |  |
| Estimated mean±se level / n |  |  |  |  |
| At 3 months | 10±1 /n=47 |  | 7±2 /n=46 |  |
| At 6 months | 11±1 /n=44 |  | 8±2 /n=43 |  |
| At 12 months | 9±1 /n=47 |  | 6±2 /n=46 |  |
| Estimated mean±se change from baseline |  |  |  |  |
| To month 3 | -2.2±0.8 | 0.006 | -2.2±0.8 | 0.007 |
| To month 6 | -1.4±0.8 | 0.09 | -1.2±0.8 | 0.13 |
| To month 12 | -3.1±0.8 | <0.001 | -3.1±0.8 | <0.001 |
| Psychological distress Score | n=190 | 0.12 | n=182 | 0.11 |
| Estimated mean±se level at baseline / n | 28±4 /n=49 |  | 24±7 /n=122 |  |
| Estimated mean±se level / n |  |  |  |  |
| At 3 months | 27±4 /n=48 |  | 24±7 /n=83 |  |
| At 6 months | 22±4 /n=45 |  | 19±7 /n=69 |  |
| At 12 months | 21±4 /n=48 |  | 17±7 /n=69 |  |
| Estimated mean±se change from baseline |  |  |  |  |
| To month 3 | -0.3±3.3 | 0.92 | 0.1±3.4 | 0.97 |
| To month 6 | -5.3±3.3 | 0.11 | -5.3±3.4 | 0.13 |
| To month 12 | -6.3±3.2 | 0.05 | -6.5±3.4 | 0.06 |

SE: Standard Error; n: number of observations. For the QLQ-C30, adjustments were made on parameters: Age, BMI, Alcoholism history, Current psychological follow-up, Alopecia and Lymphedema. For the Body Image Scale, adjustments were made on parameters: Age, BMI, Dependent child, Alcoholism history, Alopecia, and Persistent pain. For the Psychological distress, adjustments were made on parameters: Age, Level of study, Alcoholism history, Current psychological follow-up, Alopecia.

Supplementary Table 2. Time to return to work and proportion of patients returning to work at 3, 6, and 12 months from the end of treatment and from the remission consultation.

|  | From remission consultation (n=46*) | |
| --- | --- | --- |
|  | Observed | Estimated |
| Time until returned to work (days) | 259 [112-365] | NE |
| Proportion of patients returning to work |  |  |
| At 3 months | 2 (4%) | 4 [1; 17] % |
| At 6 months | 5 (11%) | 11 [5; 25] % |
| At 12 months | 8 (17%) | 30 [11; 64] % |

Data are medians [25th-75th], or number (%), or estimated % [95% Confidence Interval]. NE: Not estimable due to insufficient data or events. *1 patient returned to work before the consultation with the oncologist and 3 patients returned to work between consultation with the oncologist and remission.
